# The Role of Contextual Factors in Private Sector Engagement: A Case Study of Private Sector Contribution to COVID-19 Mitigation in Nigeria

**DOI:** 10.3389/fpubh.2022.915330

**Published:** 2022-06-21

**Authors:** Chinyere Okeke, Godstime O. Eigbiremolen, Benjamin Uzochukwu, Chinyere Mbachu, Obinna Onwujekwe

**Affiliations:** ^1^Health Policy Research Group, Department of Pharmacology and Therapeutics, College of Medicine, University of Nigeria, Enugu, Nigeria; ^2^Department of Community Medicine, College of Medicine, University of Nigeria, Enugu, Nigeria; ^3^Department of Economics, University of Nigeria, Nsukka, Nigeria; ^4^Department of Health Administration and Management, Faculty of Health Sciences and Technology, University of Nigeria, Enugu, Nigeria

**Keywords:** CACOVID, public-private partnership, COVID-19, pandemic, Nigeria

## Abstract

The Private Sector Coalition against COVID-19 (CACOVID) was established on the 27th of March 2020 to mobilize private sector resources toward supporting the government's response to the COVID-19 pandemic. More specifically, CACOVID set out to provide leadership functions, raise public awareness, provide buy-in for COVID-19 prevention, and provide direct support to strengthen the health system's capacity to respond to the crisis. In this paper, we examine the contextual factors that shaped the private sector's engagement in the fight against the pandemic with a view to identifying progress and learning opportunities. A desk review of the existing literature and documents from relevant stakeholders (government, organized private sector, and civil society organizations) was carried out. Using both the Grindle and Thomas (1) and Husted and Salazar (2) frameworks, we identified individual characteristics (industry expertise and position, philanthropy, and personal/economic interest); the economic crises created by the pandemic; a weak health system; and the multi-sectoral nature of the response to the pandemic.as contextual factors that influenced public-private collaboration in tackling the COVID-19 pandemic in Nigeria. That is, the private sector collaborated with the government based on several interrelated contexts that confront them with issues they need to address; determine what options are feasible politically, economically, and administratively; set limits on what solutions are eventually considered; and respond to efforts to alter existing policies and institutional practices. The identified contextual factors provide learning opportunities for enhancing public-private partnership in advancing healthcare not just in Nigeria, but also in related countries in Africa and other developing countries.

## Introduction

The impact of COVID-19 on the health and livelihoods of the people in low-and middle-income countries has been substantial (3). The impact was mostly felt by the lower socio-economic groups of the society. The impact of the pandemic on health led to the enactment and adoption of different policies to curb the spread of the virus (4). This had significant effects on productivity because most sectors of the economy were directly or indirectly affected by disruptions to supply chains and falling consumer demand (4).

Like many other countries in Sub-Saharan Africa, Nigeria's weak health systems, as well as political dynamics and government structures, did not allow it to respond in the most effective way when COVID-19 was first diagnosed in the country on the 27th of February 2020 (5). An organized and robust response to the pandemic was inhibited by staff shortages, weak medical supply chains, shortage of water and sanitation facilities, lack of surge capacity, and poor medical facilities that have persisted over time. These problems reflect many years of insufficient health financing and weak health governance (6). The fragile nature of Nigeria's health systems, its weak institutional capacity, and limited economic resources mean that the government needed all the support it could get.

About a month after the index case of the virus was diagnosed in Nigeria (7), the Nigerian private sector assumed a leading role in the fight against the COVID-19 pandemic in Nigeria. The Private Sector Coalition against COVID-19 (CACOVID) was established on the 27th of March 2020 to mobilize private sector resources toward supporting the government's response to the pandemic (8, 9). It is a private-sector task force in partnership with the federal government, the Nigeria Center for Disease Control (NCDC), and the World Health Organization (WHO). The sole aim of the task force is to combat COVID-19 in Nigeria (8).

The task force is composed of three leadership teams: the funding committee, the technical committee, and, the operational committee (8). The funding committee is responsible for raising funds for the activities of CACOVID and its membership includes leading entrepreneurs and business leaders in Nigeria. The members include: Godwin Emefiele, Aliko Dangote, Herbert Wigwe, Abdulsamad Rabiu, Femi Otedola, Folorunso Alakija, Jim Ovia, John Coumantaros, Raj Gupta, Segun Agbaje, Tony Elumelu, Modupe Alakija and Folorunso Alakija. The responsibility of the technical committee is to provide intellectual leadership as it concerns test-related issues, treatment protocols, management of isolation centers, etc. The members, who are mainly health professionals include Akin Abayomi, Dhamari Naidoo, Christian Happy, Phillip Onyebujo, Chikwe Ihekweazu, Paulin Basing, Zouera Youssoufou, and Omobolanle Victor-Laniyan. The operation committee is responsible for managing the daily activities of the task force and its membership includes representatives drawn from different private corporations. Key private sector corporations in the initiative include manufacturing firms (Dangote Group, BUA Group, Flour Mills of Nigeria PLC), commercial banks (Access bank, Zenith Bank, First Bank, etc.), telecommunication firms (MTN), etc.

As of 30th June 2020, CACOVID had raised about 30.2 billion Naira (about 73 million USD) through donations from members of the task force as well as other individuals (philanthropists) and business enterprises (8). See Appendix A for a detailed list of contributors to the CACOVID relief fund with the amount contributed. The funds raised were used to finance the establishment of treatment centers (establishment of medical facilities in the six geopolitical zones of the country), the establishment of testing centers (optimizing diagnostic capacity for COVID-19 testing), training of health personnel, the building of isolation facilities across the country, food relief programme across different States, the provision of household essentials, and the provision of conditional cash transfers to support the many households that were and are still grappling with the economic hardships imposed by the pandemic.

Although the usefulness of the private sector involvement in the fight against the COVID-19 pandemic in Nigeria is widely acknowledged (9), little is known about the contextual factors that influenced their engagement. The need to fill this gap in knowledge is the primary aim of this study. Understanding these factors will help to build a resilient and sustainable private sector partnership that will persist into the future and last beyond tackling the pandemic. The findings from this study will be useful to policymakers, civil society organizations, the private sector themselves, and other sectors, both in times of pandemics and other times.

## Materials and Methods

### Description of Study Area

Nigeria is still ranked among the poorest countries in the world, with about 70% of the population living below US$1 per day. About 52.2% of the country's population live in rural areas where poverty is more predominant, thus limiting access to adequate nutrition, quality health care, and other basic social services. The health care system is largely public sector driven, with substantial private sector involvement in service provision. Secondary- and tertiary-level health facilities are mostly found in urban areas, whereas rural areas are predominantly served by primary health care (PHC) facilities. There is a shortage of PHC facilities in some states. Health policy-making and national health care priority setting are the responsibility of the federal government.

### Analytical Framework

To guide our analysis and discussions, we drew on theories, frameworks, and concepts in the literature. Grindle and Thomas (1) conceptualize context as including the structure of a class and interest group mobilization in the society, historical experiences and conditions, economic and political relationships, domestic economic conditions, the administrative capacity of the state, and the impact of prior or conterminously pursued policies. They also include in a context, the individual characteristics of policy actors such as their ideological predispositions, professional expertise, and training, memories of similar policy situations, position and power resources, political and institutional commitments, loyalties, and personal attributes and goals. They observe that policy actors (i.e., anyone who influences policymaking, including private individuals, corporations, and other non-state actors) are never fully autonomous. Instead, they work within several interlocking contexts that confront them with issues and problems they need to address, set limits on what solutions are considered, determine what options are feasible politically, economically, and administratively, and respond to efforts to alter existing policies and institutional practices. Contextual factors, which are defined to remain the same in a given context, may serve as a source of power to influence actors' actions, inaction, and choice. This power, which is mostly in the form of soft power that is expressed through consent rather than force, is derived primarily from actors' reputation, expertise, economic positions, skills, etc. Actors therefore can become influencers within a specific context to affect intervention processes. As noted by Mintzberg (10), to be an influencer, one requires some authority, coupled with active involvement in ongoing processes in a skillful way.

### Study Design

A descriptive case study design was used to explore the contextual factors that contributed to the private sector engagement and contribution to COVID-19 mitigation in Nigeria. The private sector was purposively selected based on evidence that they (i.e., the private sector) contribution was significant and that a lot of contextual factors shaped the response of CACOVID. These contextual factors were identified through desk review and the Grindle and Thomas (1) framework and selected based on their relevance to the fight against the COVID-19 pandemic. The contextual factors include (i) individual characteristics such as industry expertise and position, philanthropy, and personal/economic interest; (ii) the economic crises created by the pandemic; (iii) a weak health system; and (iv) the multi-sectoral nature of the response to the pandemic.

### Data Collection Method and Data Analysis Method

A desk review of literature and articles from stakeholders (government, organized private sector, and CSOs) was carried out^1^. Programme documents and published research articles were sourced from Scopus, PubMed, Google scholar, and Directory of Open Access databases. Government documents were retrieved electronically from organizational websites. Articles published in the English language between January 2020 and 2021 were retrieved using a combination of keywords coined to retrieve materials peculiar to the private sector CACOVID. They include: (“COVID-19” and/or “coronavirus” and/or “lockdown” and/or “government” and/or “response” and/or “palliatives” and/or “Enugu City” and/or “CSOs” and/or “urban” and/or “urban group” and/or “disabilities” and/or “private sector” and/or “CACOVID”). Data were extracted from each document using specific themes as stated above. The template was prepared in Microsoft Excel. Retrieved sources were critically read to identify and document significant findings pertaining to contextual issues that relate to private sector functions in the mitigation of COVID-19. Relevant information from each document was carefully summarized/paraphrased and entered into the excel file. Data gotten were analyzed in themes that reflect responses at the national levels and the various components of the framework being used for this study. We retrieved 52 articles but only 14 were included based on the set criteria (4 peer-reviewed articles, 1 blog, 7 reports and briefs on development partners' websites, and 2 gray literature). However, we included and cited external sources, especially in the introduction and discussion to provide general background on Nigeria's health system and economy.

## Results

Context and actors consistently influenced the manner in which private sector actors intervened in the response to the pandemic in Nigeria. The identified factors include individual characteristics and interests; the economic crisis created by the pandemic; health outcomes; international participation; and the multi-sectoral nature of the response to the pandemic.

### Individual Characteristics and Interests

The individual characteristics and interests that influenced the involvement of the key stakeholders behind the private initiative to support the government's fight against the COVID-19 pandemic (CACOVID) are presented below.

#### Industry Expertise and Position

Most of the key stakeholders of CACOVID are key industry players in Nigeria. They play leading roles in the economy and have substantial positions and power in society. Among them are the leading captains of industry and the wealthiest entrepreneur in Nigeria and the African continent (11). They represent the upper class of Nigerian society. This position comes with power and influence, especially in a developing country like Nigeria (12). Their unique position makes it possible for them to not only make a substantial financial contribution to the private sector initiative but also to leverage their high network to raise additional funds to support the fight against the pandemic. For example, a key member of the task force and the richest man in Africa, Aliko Dangote, contributed 2 billion Naira (about 5 million USD) to the relief fund through Dangote industries limited. Apart from making substantial donations and facilitating fund mobilization, the key stakeholders of the private sector initiative are also individuals with years of industry experience. This experience was brought to bear to ensure that the activities of CACOVID remained aligned with its goals. Thus, the industry expertise and position occupied by the private sector key stakeholders are a major determinant of their engagement and support in the fight against the pandemic.

#### Philanthropy

This is described in the entrepreneur-philanthropy model, which opined that successful entrepreneurs are often inclined to make a philanthropic investment that is specifically aimed at addressing a targeted societal problem (13). The key stakeholders of the private sector initiative against the COVID-19 pandemic in Nigeria are well-established entrepreneurs with business interests across different sectors of the economy. These characteristics, in line with the entrepreneur-philanthropy nexus espoused by Acs and Phillips (14), predispose the private sector leaders to use their resources for the greater good of society. This is consistent with the theory of corporate social responsibility, which notes that private businesses and establishments have philanthropic obligations to the society wherein they operate (13).

#### Personal/Economic Interest

The characteristics of the private sector leaders that we have considered so far suggest that their decision to support the government in its fight against the COVID-19 pandemic is purely altruistic. This may not be the case since they have their interest to protect as well. That is, the private sector stakeholders have a direct stake or benefit in curtailing the outbreak of the pandemic in Nigeria (9, 15–17). As noted earlier, the private sector leaders have expansive business interests across sectors in Nigeria. If not curtailed, the pandemic could cost them a lot. Thus, as they are helping to fight the pandemic, they are also helping to preserve their businesses. Even if we argue that the key partners of CACOVID have large corporations that are resilient with extensive technological and logistical capabilities to keep their business activities ongoing in the face of the pandemic, the vast majority of smaller businesses that are invaluable in their value chain do not have such capabilities to withstand the pandemic (9). These smaller businesses consist of a collection of micro, small and medium enterprises (MSMEs) from the smallholder farmers who supply agricultural inputs to food processing conglomerates, to the street vendors who distribute products for the fast-moving consumer goods sector. Thus, anything that affects these smaller businesses will also affect their own businesses.

In addition, the key private sector leaders are not oblivious of the fact that their decision to partner with the government in combating the pandemic will generate corporate goodwill for them. This goodwill, which is an intangible asset, can serve to differentiate their businesses from that of their competitors, which will provide an opportunity to generate additional economic profits.

### The Economic Crisis Created by the Pandemic

Another contextual factor that influenced the private sector's continual engagement and partnership with the government is the huge economic crises created by the pandemic. Like many other countries of the world, the Nigerian Government enforced lockdown measures in March 2020 in a bid to control the spread of the virus. The lockdown and other COVID-19 restrictions eroded the already fragile access to food and livelihood for many Nigerians (18, 19). Unlike in many developed countries, the absence of a functioning social security system means that Nigerians were particularly vulnerable to the economic impact of the COVID-19 pandemic (18). For example, developed countries have existing social safety programmes like unemployment benefits, child benefits, cash transfers, food banks, etc. that are designed to cushion the effect of shocks like the pandemic. These countries also developed additional specific social support measures to cushion the negative economic effects the pandemic has on the vulnerable members of the society (20, 21). All these social protection interventions were largely missing in Nigeria and households were left to fend for themselves. In its 2019 report, the World Bank noted that only about 4 percent of households among the poorest 40 percent have access to any form of social safety net programmes (22).

Falling incomes due to the restriction of movement imposed by the government, rising food prices, and little or no government support means an increased hunger in the country (18). A survey conducted by the National Bureau of Statistics (NBS) in 2020 found that many households ran out of food in the past.

**Figure 1:**
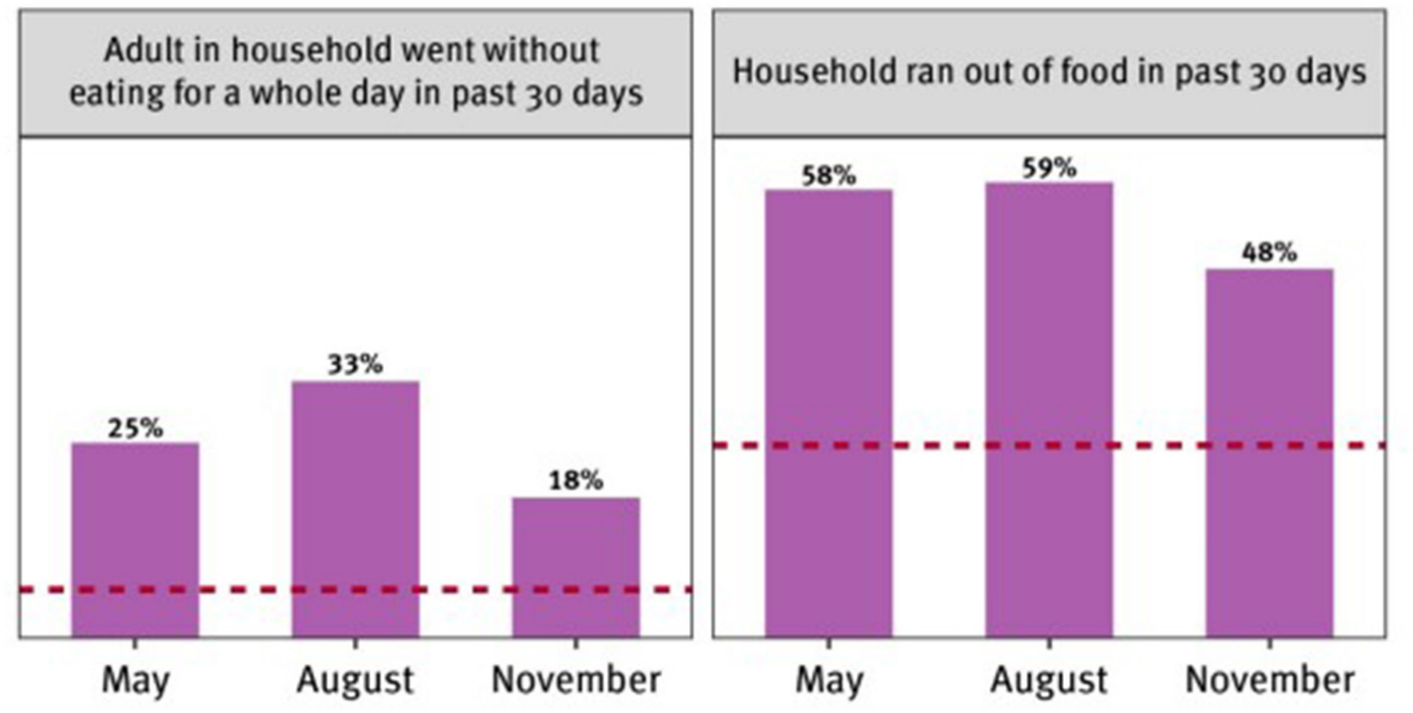
Hunger during the pandemic. Source: Human Right Watch analysis of the NBS 2020 COVID-19 phone survey.

Thirty days of the survey and that adults in some households were going without eating food for a whole day (Figure 1). Comparing the current values to the baseline values (the red lines represent 2018–2019 baseline values) suggests that hunger has more than doubled as a result of the pandemic, “ceteris paribus.”

**Figure 2:**
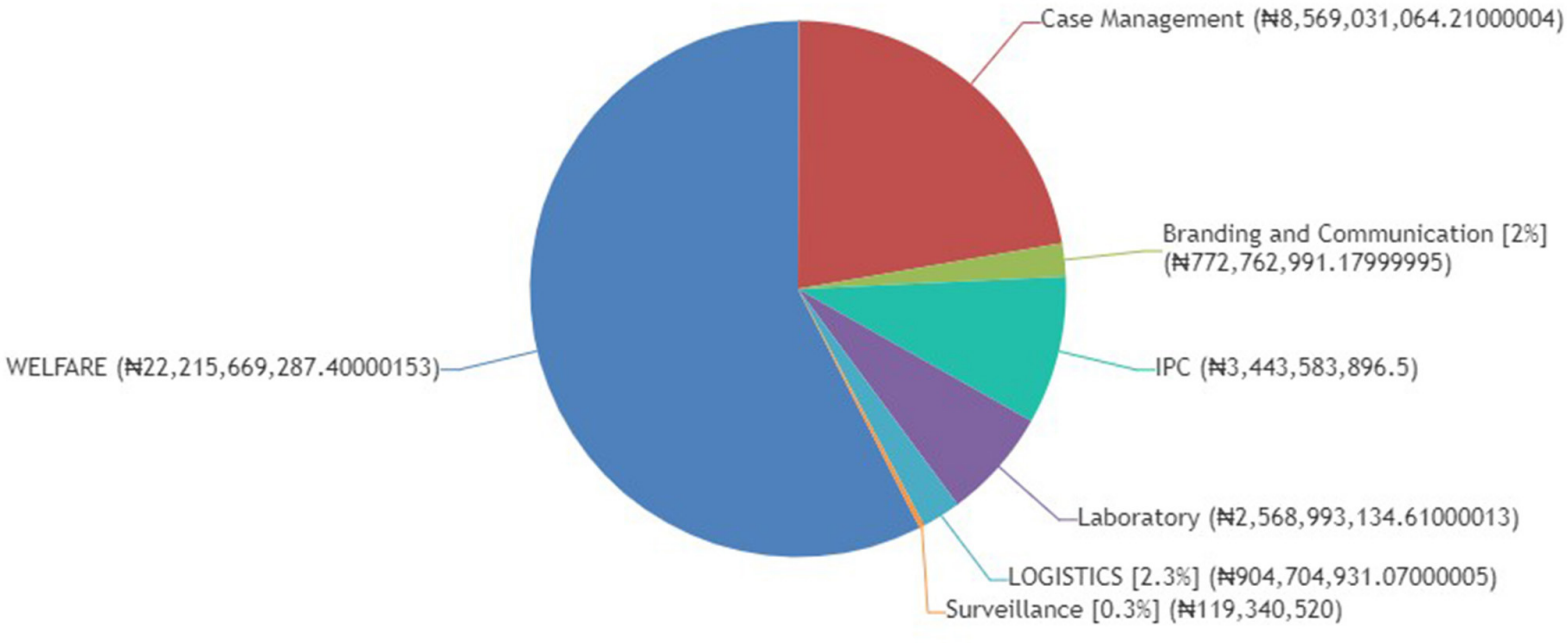
CACOVID expenditure. Source: www.cacovid.com.

A look at the distribution of CACOVID's total expenditure for the year ending 2020 in Figure 2 makes this point very clear. More than half (about 57%) of the total intervention cost was spent on welfare. These were mostly in the form of food relief programme across different States, household essentials, and conditional cash transfers to support the many households that were and are still grappling with the economic hardships imposed by the pandemic.

### Weak Health System

The weakness of Nigeria's health system is another contextual factor that influenced the private sector's engagement in the fight against the pandemic. The country has been ranked 163 out of 191 countries in WHO's health system ranking of November 2021 (23). She was also placed at 142 out of 195 countries according to a Lancet report's ranking of health systems performance using healthcare access and quality as its criteria (24) and ranks poorly based on the World Bank's Universal Health Coverage Service Coverage Index (25). Recent assessments have shown that the maternal mortality ratio is 512 per 100 000 live births, the under-5 mortality rate is 132 per 1,000 live births, the infant mortality rate is 67 per 1,000 live births, and life expectancy is 52.62 years (26). A lot of children were wasted and stunted with percentages estimated at 18 and 37%, respectively and only 46% of deliveries were attended by Skilled Birth Attendants (26). In addition, universal child vaccination has remained a public health challenge in Nigeria, despite some improvements recorded in vaccination uptake between 2008 and 2020 (27). For example, in 2018, only 31% of children aged 12–23 months received all primary vaccinations, only 28% received the basic vaccinations by the appropriate age of 12 months, and as many as 19% received no vaccinations at all (28). These are core equity indicators for measuring a country's performance and it is clear that Nigeria was not meeting up expected health outcomes.

These poor health indicators were made worse by the COVID-19 pandemic. As of 30th April 2022, Nigeria had recorded 255,759 cases, 249,911 discharges, 3,143 deaths, and 2,699 active cases (29). The poor turn-around time for COVID-19 test results and the huge stigma associated with the disease at the onset served as motivation for the organized private sector's fast involvement (30, 31). The huge out-of-pocket expenses for health care in the country as well as the inability of most Nigerians to earn money because of measures instituted to contain the pandemic led many Nigerians to become economically impoverished by the COVID-19 pandemic and so cannot afford health care (32). The increased hospitalization associated with the COVID-19 pandemic is over-stretching the resilience of the health system of most countries, especially those of low- and middle-income countries, and the health workers are overwhelmed by the numbers of people requesting testing and treatment at the same time (33). COVID-19-related commodity procurement was least responsive to the needs of those most in need of care and support (23).

The government then instituted several fiscal policies to improve funding for the health sector due to the impact of COVID-19. One of the immediate responses to ease the financial impact of COVID-19 is the inclusion of COVID-19 management in health insurance packages and an increase in domestic government health spending to at least 5% of gross domestic product (32). However, the long years of neglect of the health system in Nigeria make it unprepared to meet the demands that COVID-19 has placed on it. Hence the appreciation of the efforts of the organized private sector, whose support helps in strengthening the health system by providing treatment centers (establishment of medical facilities), the establishment of testing centers, the training for health personnel, and the building of isolation facilities.

### Multi-Sectoral Nature of the Response to the Pandemic

It was identified early that one of the solutions to the pandemic lies in collective strength, cooperation, and good leadership at all levels. Fighting a pandemic requires a multinational approach tailored toward local realities. Thus, one of the keys to effective containment is partnership and collaboration. This is where the private sector comes in. Apart from financial contribution, the private sector can also drive community engagement, communication, procurement, and even the manufacturing of drugs and equipment. Urgent and multi-sectoral efforts were needed to stop the exportation of the disease and to help with the diagnosis and treatment aspect (34). It was also borne out of fear that the disease will blow up quickly beyond the capacity of the country to handle it and that the health system will be overwhelmed to the point of collapse (34). It was, therefore, necessary for the private sector to cooperate with the government and join in the fight against the pandemic. The private sector, collaborated with the government through the leadership of its task force that comprises entrepreneurs and business leaders, health professionals, and administrators.

## Discussion

**Figure 3:**
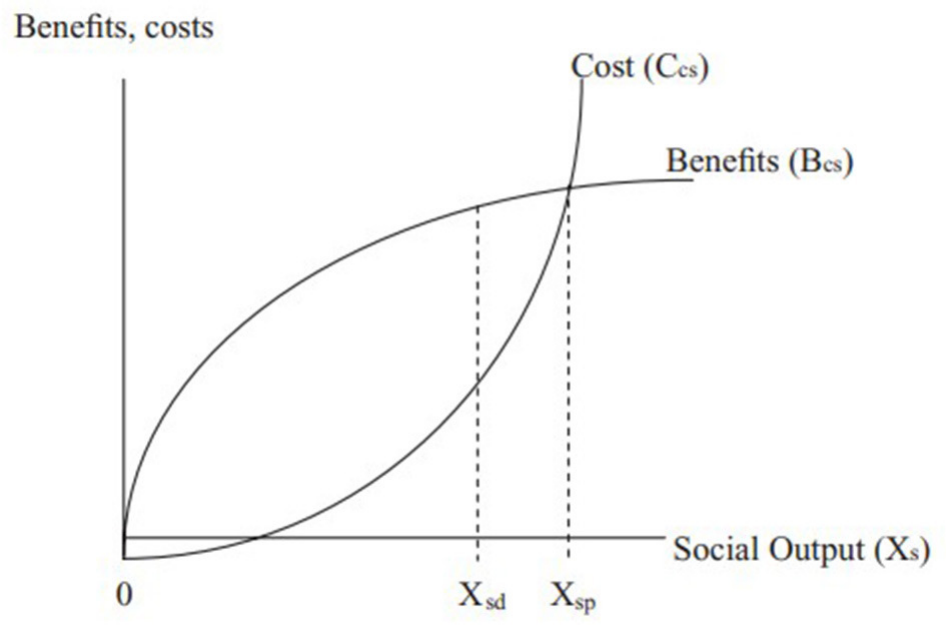
Optimum social investment in the case of altruism. Source: Husted and Salazar (2).

This study examined contextual factors that influence private sector engagement with a view to provide an understanding of this area and identify where progress is being made that offers opportunities for learning. Our findings show that although the primary reason why CACOVID's key leaders intervened may be primarily altruistic, there is an element of strategic behavior at play that see them also benefiting from their intervention. That is, the private sector engagement is the case of altruism with a touch of strategic behavior or self-interest. This is better explained using the Husted and Salazar (2) optimal social investment model in the case where altruism is the primary driving force. The Husted and Salazar (2) framework serves as an expansion and as a supporting framework to the Grindle and Thomas (1) framework in our context. The model is presented in Figure 3 and the discussion is restricted to the basics of the model.

Social output (X_s_), which is represented in the horizontal axis of Figure 3, measures the contribution or benefit to society as a result of the CA-COVID partnership with the government in combating the pandemic. It measures the positive externality that emanates from their intervention. This benefit could be in the number of deaths averted and the number of businesses and jobs preserved that could have been lost to the pandemic. The private sector faces a social cost curve (Ccs), which represents the total amount of money spent for every given level of social benefit created. That is, the social cost curve shows the cost to the private sector of producing an additional unit of social output.

In line with standard microeconomic analysis, the model assumes that the cost to the private sector obeys the law of diminishing marginal returns. That is, for each additional unit of social output produced by the private sector, the cost advantage diminishes. The intuition here is that we expect the first units of social output to be relatively inexpensive. These are like low-hanging fruits that are easy to pluck. For example, awareness creation about the effect of COVID-19 that emphasizes a change in behavior (e.g., the use of facemasks, hand washing, social distancing, etc.) could generate a significant amount of social output at a relatively low cost. However, as additional units of social output are created (everyone in the society will not change their behavior toward the virus, hence the need to produce more social output), the cost to the private sector will increase (33). This extra effort may include building and equipping hospitals, purchasing ventilators, etc. This explains the upward-sloping nature of the cost curve. This process will continue until social output gets to Xsp. In our case, Xsp can be likened to the complete eradication of the COVID-19 virus in Nigeria.

The discussion of the model so far is solely altruistic: the private sector incurs costs in order to create social output or mitigate a problem in society (i.e., the COVID-19 pandemic in our case). However, the model represented in Figure 3 indicates that apart from the cost incurred and the benefit generated to society, the private sector also receives a benefit. This is represented by the benefit curve (Bcs), which is a private benefit that is different from the social benefit. The benefit curve, therefore, captures the gains that accrue to the private sector for every unit of social output produced. Put differently, the benefit curve represents private returns to social investment. These benefits could be in the form of increased sales due to corporate goodwill that follows their intervention or a better relationship with the government, both of which could lead to increased revenue. The altruistic private sector leaders that chose to invest their resources for the good of society are ab initio aware of this benefit (2).

Private sector actors acted within interrelated contextual factors. Our observations are in keeping with similar observations by Grindle and Thomas (1), that contextual factors working in interrelating manner can serve as a constraint and an opportunity within which policy actors maneuver to accomplish their goals. The government of Nigeria instituted several fiscal policies to improve funding of the health sector due to the impact of COVID-19 such as taxing diaspora remittances; swopping debt reduction for domestic investment in health systems; auctioning or sale of emissions permits; trading of Special Drawing Rights; an effective collection of corporate and business taxes; and addressing cross-border tax fraud, evasion, and avoidance (35). This was similarly seen in other settings, especially low and middle-income countries like Kenya (36). These were seen to attract the organized private sector to function well.

The private sector linkages were built to grow the compliance rate of the residents with safety measures, increase the provision of palliatives for low income groups, and add to the medical and non-pharmaceutical resources of the government which is key to defeating the pandemic. Similar efforts were made by the governments of many developed countries. But in the case of Nigeria, the government was overstretched and could not provide for all the needs of its large population. Hence, the intervention by the organized private sector.

## Conclusion

COVID-19 pandemic is quickly reshaping our lives, economies, and health care systems. The intervention of the private sector through CACOVID was a timely and a welcomed strategy to save the country from the more devastating effects of the pandemic. As of 30th June 2020, CACOVID had raised about 30.2 billion Naira (about 73 million USD) through donations from members of the task force as well as other individuals (philanthropists) and business enterprises (8). The funds raised were used to finance the establishment of treatment centers, the establishment of testing centers, the training of health personnel, the building of isolation facilities across the country, food relief programme across different States, the provision of household essentials, and the provision of conditional cash transfers to support the many households that were and are still grappling with the economic hardships imposed by the pandemic. The identified contextual factors that influenced the engagement of the private sector and the establishment of CACOVID include individual characteristics such as industry expertise and position, philanthropy, and personal/economic interest; the economic crises created by the pandemic; a weak health system; and the multi-sectoral nature of the response to the pandemic. Put differently, the private sector collaborated with the government based on several interrelated contexts that confront them with issues they need to address; determine what options are feasible politically, economically, and administratively; set limits on what solutions are eventually considered; and respond to efforts to alter existing policies and institutional practices. The identified contextual factors provide learning opportunities for enhancing public-private partnership in advancing healthcare not just in Nigeria, but also in related countries in Africa and other developing countries.

## Data Availability Statement

The original contributions presented in the study are included in the article/Supplementary Material, further inquiries can be directed to the corresponding author.

## Author Contributions

CO and GE: review and drafting. BU, CM, and OO: conceptualization/design. All authors contributed to the article and approved the submitted version.

## Funding

This study is part of the Community-led Responsive and Effective Urban Health Systems (CHORUS) funded by UK Aid, from the UK Government, Grant 301132.

## Conflict of Interest

The authors declare that the research was conducted in the absence of any commercial or financial relationships that could be construed as a potential conflict of interest.

## Publisher's Note

All claims expressed in this article are solely those of the authors and do not necessarily represent those of their affiliated organizations, or those of the publisher, the editors and the reviewers. Any product that may be evaluated in this article, or claim that may be made by its manufacturer, is not guaranteed or endorsed by the publisher.

## Footnotes

^1^This is part of the Community-led Responsive and Effective Urban Health Systems (CHORUS) project.

## References

[1] Grindle MS Thomas JW. Public Choices and Policy Change: the Political Economy of Reform in Developing Countries. Baltimore, MD: Johns Hopkins University Press (1991).

[2] Husted BW de Jesus Salazar J. Taking Friedman seriously: maximizing profits and social performance. J Manag Stud. (2006) 43:75–91. 10.1111/j.1467-6486.2006.00583.x

[3] Evans D, Over, M,. The Economic Impact of COVID-19 in Low- Middle-Income Countries. (2020). Available online at: https://www.cgdev.org/blog/economic-impact-covid-19-low-and-middle-income-countries

[4] Ohrnberger J Segal AB Forchini G Miraldo M Skarp J Nedjati-Gilani G. The impact of a COVID-19 lockdown on work productivity under good and poor compliance. Eur J Public Health. (2021) 31:1009–15. 10.1093/eurpub/ckab138 34358291 PMC8385936

[5] Health Minister: First Case of COVID-19 Confirmed in Nigeria. Available online at: https://www.health.gov.ng/index.php?option=com_k2&view=item&id=613:health-minister-first-case-of-covid-19-confirmed-in-nigeria

[6] Olu O Drameh-Avognon P Asamoah-Odei E Kasolo F Valdez T Kabaniha G. Community participation and private sector engagement are fundamental to achieving universal health coverage and health security in Africa: reflections from the second Africa health forum. BMC Proc. (2019) 13(Suppl 9): 7 10.1186/s12919-019-0170-0 PMC6849158 31737089

[7] Ebenso B Otu A. Can Nigeria contain the COVID-19 outbreak using lessons from recent epidemics? Lancet Glob Health. (2020) 8:e770. 10.1016/S2214-109X(20)30101-7 32171055 PMC7104043

[8] Available, online at: www.cacovid.org

[9] United Nations (2020),. The Private Sector's Role in Mitigating the Impact of Covid-19 on Vulnerable Women and Girls in Nigeria. Available online at: https://www.weps.org/sites/default/files/2020-04/Nigeria_PrivateSector_Brief_v4COVID19_0.pdf (accessed October 7, 2021).

[10] Mintzberg H. Power in and Around Organizations. New Jersey: Prentice-Hall (1983).

[11] Forbes. The Forbes Billionaires' List: Africa's Richest People 2021. (2021). Available online at: https://www.forbes.com/sites/kerryadolan/2021/01/22/the-forbes-billionaires-list-africas-richest-people-2021/?sh=74522de748f5 (accessed October 8, 2021)

[12] Kerbo H,. Power in Modern Societies. (1993). Available online at: https://www.taylorfrancis.com/chapters/edit/10.4324/9780429302824-23/upper-class-power-harold-kerbo (accessed October 7, 2021).

[13] Acs ZJ Desai S. Democratic capitalism and philanthropy in a global economy. Jena Economic Research Paper. (2007) 25:2–28. 10.2139/ssrn.1022921

[14] Acs ZJ Phillips RJ. Entrepreneurship and philanthropy in American capitalism. Small Business Economics. (2002) 19:189–204. 10.1023/A:1019635015318

[15] Impacts of COVID-19 on the Private Sector in Fragile and Conflict-Affected Situations. Available online at: https://reliefweb.int/sites/reliefweb.int/files/resources/EMCompass_Note%2093-COVID%20and%20FCS_Nov2020.pdf

[16] Understanding the impact of the COVID-19 outbreak on the Nigerian economy. Available online at: https://www.brookings.edu/blog/africa-in-focus/2020/04/08/understanding-the-impact-of-the-covid-19-outbreak-on-the-nigerian-economy/

[17] The Impact of COVID-19 on Business Enterprises in Nigeria. Available online at: https://www.ng.undp.org/content/nigeria/en/home/library/mdg/the-impact-of-covid-19-on-business-enterprises-in-nigeria.html

[18] Human Right Watch (2021). Available at https://www.hrw.org/news/2021/07/28/nigeria-covid-19-impact-worsens-hunger-lagos

[19] Balana BB Oyeyemi MA Ogunniyi AI Fasoranti A Edeh H Aiki J. The effects of COVID-19 policies on livelihoods and food security of smallholder farm households in Nigeria: descriptive results from a phone survey. IFPRI. (2020) 34. 10.2499/p15738coll2.134179

[20] Who's helping me? Perceptions of social safety nets during COVID-19. Available online at: https://www.oecd-forum.org/posts/who-s-helping-me-perceptions-of-social-safety-nets-during-covid-19

[21] Valente. Safety Nets: An International Comparison. (2019). Available online at: https://newlaborforum.cuny.edu/2019/06/05/safety-nets-an-international-comparison/

[22] World Bank. Advancing Social Protection in a Dynamic Nigeria (2019). Available online at: https://documents1.worldbank.org/curated/en/612461580272758131/pdf/Advancing-Social-Protection-in-a-Dynamic-Nigeria.pdf (accessed October 8, 2021).

[23] WHO Health Systems Performance Ranking For Nigeria Geneva: World Health Organization (2021).

[24] Fullman N Yearwood J Abay SM Abbafati C Abd-Allah F Abdela J. Measuring performance on the Healthcare Access and Quality Index for 195 countries and territories and selected subnational locations: a systematic analysis from the Global Burden of Disease Study 2016. Lancet. (2018) 391:2236–71. 10.1016/S0140-6736(18)30994-2 29893224 PMC5986687

[25] World Bank. UHC Service Coverage Index-Nigeria Washington DC, The World Bank. (2017).

[26] NPC N. Nigeria Demographic and Health Survey 2018-Final Report, Dhsprogram.com. Available online at: https://dhsprogram.com/publications/publication-fr359-dhs-final-reports.cfm

[27] Otoimo L, Abata, S,. Factors Associated with the Change in Vaccination Uptake in Nigeria: A Decomposition Analysis. (2021). Available online at https://ipc2021.popconf.org/uploads/211042

[28] National Population Commission (NPC) [Nigeria] and ICF. Nigeria Demographic and Health Survey 2018. NPC and ICF (2019).

[29] COVID-19 in Nigeria. Available online at: https://covid19.ncdc.gov.ng/

[30] All Africa. Outcry as Nigerians wait for one week to get COVID-19 results (2020). Available online at: https://allafrica.com/stories/202005110228.html [Ref list]

[31] Healthnews.ng. Nigeria's covid-19 response threatened by social stigma (2020). Available online at: http://www.healthnews.ng/nigerias-covid-19-response-threatened-by-social-stigma/ [Ref list]

[32] Aregbeshola BS Folayan MO. Nigeria's financing of health care during the COVID-19 pandemic: Challenges and recommendations. World Medical & Health Policy, (2021).14:1–10. 34909238 10.1002/wmh3.484 PMC8661625

[33] Nigeria Health Watch. “At the frontlines of Nigeria‘s COVID-19 response: The laboratory.” Available online at: https:nigeriahealthwatch.com-at-the-frontlines-of-nigerias-covid-19-response-thelaborator/#.XobfTsoo84N

[34] Los Angeles Times. “What happens if the coronavirus outbreak becomes a pandemic?” Available online at:https://www.latimes.com/science/story/2020-02-05/coronavirus-pandemic-preparations

[35] Qui W Rutherford S Mao A Chu C. “The pandemic and its impacts.” Health, Culture and Society (2017). p. 9–10. Available online at: https://hcs.pitt.edu/ojs/index.php/hcs/article/view/221 10.5195/HCS.2017.221

[36] Diakonia, OxfamTI. Urgent Call for Debt Relief, Transparency and Accountability in Management of COVID-19 Emergency Funds to Ensure they Respond to the Needs of the People Affected. Available online at https://reliefweb.int/report/kenya/urgent-call-debt-relief-transparency-and-accountability-management-covid-19-emergency (accessed 8 May, 2020).

