# Supplementary material for: The Role of Contextual Factors in Private Sector Engagement: A Case Study of Private Sector Contribution to COVID-19 Mitigation in Nigeria
Source: Front Public Health. 2022 Jun 21;10:915330. doi: 10.3389/fpubh.2022.915330 (PMC9253585; doi:10.3389/fpubh.2022.915330)
Supplement: Supplementary file 1 [file Table_1.pdf]

APPENDIX A: TABLE 1: LIST OF CONTRIBUTORS TO THE CACOVID RELIEF FUND as of 30 June 2020

S/N	Name	Company	Amount (Naira)
1	CENTRAL BANK OF NIGERIA	CENTRAL BANK OF NIGERIA	2,000,000,000.00
2	ALIKO DANGOTE	DANGOTE INDUSTRIES LIMITED	2,000,000,000.00
3	FLOOD RELIEF FUND	PRESIDENTIAL COMMITTEE ON FLOOD RELIEF AND REHABILITATION	1,500,000,000.00
4	ABDULSAMAD RABIU	BUA SUGAR REFINERY LIMITED	1,000,000,000.00
5	FEMI OTEDOLA	AMPERION POWER DISTRIBUTION LTD	1,000,000,000.00
6	HERBERT WIGWE	ACCESS BANK PLC	1,000,000,000.00
7	JIM OVIA	ZENITH BANK PLC	1,000,000,000.00
8	JOHN COUMANTATOUS	FLOUR MILLS OF NIGERIA PLC	1,000,000,000.00
9	MIKE ADENUGA	MIKE ADENUGA FOUNDATION	1,000,000,000.00
10	MODUPE & FOLORUNSHO +JA	FAMFA OIL LIMITED	1,000,000,000.00
11	MTN NIGERIA PLC	MTN NIGERA PLC	1,000,000,000.00
12	NDIC	NIGERIA DEPOSIT INSURANCE CORPORATION	1,000,000,000.00
13	OBA OTEDEKO	FIRST BANK OF NIGERIA	1,000,000,000.00
14	RAJ GUPTA	AFRICAN STEEL MILLS NIG. LTD	1,000,000,000.00
15	SEGUN AGBAJE	GUARANTY TRUST BANK PLC	1,000,000,000.00
16	TONY ELUMELU	UNITED BANK OF AFRICA	1,000,000,000.00
17	OLAM NIGERIA LIMITED	OLAM NIGERIA LIMITED	600,000,000.00
18	INDORAMA ELEME FERTILIZER CHEMICAL	INDORAMA ELEME FERTILIZER CHEMICAL	550,000,000.00
19	UNITED BANK FOR AFRICA PLC	UNITED BANK FOR AFRICA PLC	500,000,338.75
20	BANK OF INDUSTRY	BANK OF INDUSTRY	500,000,000.00
21	DEJI ADELEKE	PACIFIC HOLDING LIMITED	500,000,000.00
22	FRIESLAND CAMPINA WAMCO	FRIESLAND CAMPINA WAMCO	500,000,000.00
23	RAHUL SAVARA	WACOT RICE LIMITED	500,000,000.00
24	TOLARAM AFRICA ENTERPRISE LTD	TOLARAM AFRICA ENTERPRISE LTD	500,000,000.00
25	DE UNITED	DE UNITED	500,000,000.00
26	RAMESH KASANGRA	NIPCO PLC	300,000,000.00
27	AFRICA FINANCE CORPORATION	AFRICA FINANCE CORPORATION	250,000,000.00
28	CITIBANK NIGERIA LTD	CITIBANK NIGERIA LTD	250,000,000.00
29	ECOBANK PLC	ECOBANK PLC	250,000,000.00
30	FCMB	FCMB	250,000,000.00
31	FIDELITY BANK PLC	FIDELITY BANK PLC	250,000,000.00
32	STANBIC IBTC	STANBIC IBTC	250,000,000.00
33	STANDARD CHARTERED BANK	STANDARD CHARTERED BANK	250,000,000.00
34	STERLING BANK PLC	STERLING BANK PLC	250,000,000.00
35	UNION BANK PLC	UNION BANK PLC	250,000,000.00

36	GILBERT CHAGOURY	GILBERT CHAGOURY	250,000,000.00
37	RONALD RAMEZ CHAGOURY	RONALD RAMEZ CHAGOURY	250,000,000.00
38	MULTICHOICE NIGERIA LIMITED	MULTICHOICE NIGERIA LIMITED	200,000,000.00
39	PROMASIDOR NIGERIA LTD	PROMASIDOR NIGERIA LTD	200,000,000.00
40	APM TERMINALS APAPA LIMITED	APM TERMINALS APAPA LIMITED	150,000,000.00
41	CORONATION MERCHANT BANK	CORONATION MERCHANT BANK	100,000,000.00
42	FBN MERCHANT BANK	FBN MERCHANT BANK	100,000,000.00
43	FSDH	FSDH	100,000,000.00
44	HERITAGE BANK	HERITAGE BANK	100,000,000.00
45	KC GAMING NETWORKS LTD	KC GAMING NETWORKS LTD	100,000,000.00
46	KEYSTONE BANK	KEYSTONE BANK	100,000,000.00
47	NOVA MERCHANT BANK	NOVA MERCHANT BANK	100,000,000.00
48	POLARIS BANK	POLARIS BANK	100,000,000.00
49	PORTS AND TERMINAL MULTISERV LTD	PORTS AND TERMINAL MULTISERV LTD	100,000,000.00
50	PROVIDUS BANK	PROVIDUS BANK	100,000,000.00
51	RAND MERCHANT BANK	RAND MERCHANT BANK	100,000,000.00
52	SUNTRUST BANK	SUNTRUST BANK	100,000,000.00
53	UNITY BANK	UNITY BANK	100,000,000.00
54	WEMA BANK	WEMA BANK	100,000,000.00
55	DEVELOPMENT BANK OF NIGERIA	DEVELOPMENT BANK OF NIGERIA	100,000,000.00
56	TIN-CAN ISLAND TERM LTD	TIN-CAN ISLAND TERM LTD	100,000,000.00
57	FPCNL PENOP AFIS ESCROW ACCT	FPCNL PENOP AFIS ESCROW ACCT	100,000,000.00
58	IHS NIGERIA LIMITED	IHS NIGERIA LIMITED	100,000,000.00
59	UAC	UAC	99,999,838.75
60	FIVE STAR LOGISTICS LIMITED	FIVE STAR LOGISTICS LIMITED	75,000,000.00
61	PORTS AND CARGO HANDLING SERV	PORTS AND CARGO HANDLING SERV	75,000,000.00
62	ENL CONSORTIUM	ENL CONSORTIUM	70,000,000.00
63	JOSEPDAM PORTS SERVICES NIG	JOSEPDAM PORTS SERVICES NIG	60,000,000.00
64	DAMTAQ LIMITED	DAMTAQ LIMITED	59,924,679.50
65	UBA	UBA	54,365,859.94
66	SYSTEMSPECS LIMITED	SYSTEMSPECS LIMITED	50,000,161.25
67	ATIKU ABUBAKAR	ADAMA BEVERAGES LIMITED	50,000,000.00
68	GLOBUS BANK	GLOBUS BANK	50,000,000.00
69	PASTOR W. F. KUMUYI	DEEPER CHRISTIAN LIFE MINISTRY	50,000,000.00
70	PORTS AND TERMINAL	PORTS AND TERMINAL	50,000,000.00

71 TAKAGRO CHEMICALS LTD	TAKAGRO CHEMICALS LTD	50,000,000.00
72 TITAN TRUST BANK	TITAN TRUST BANK	50,000,000.00
73 WA CONTAINER TERMINAL	WA CONTAINER TERMINAL	50,000,000.00
74 ADEREMI MAKANJUOLA	ADEREMI MAKANJUOLA	50,000,000.00
75 PREMIER LOTTO LIMITED	PREMIER LOTTO LIMITED	49,999,838.75
76 KAM WIRE LIMITED	KAM WIRE LIMITED	30,000,000.00
77 DE DAMAK NIG LTD AUTOMOBILE	DE DAMAK NIG LTD AUTOMOBILE	25,000,000.00
78 ADRON HOMES PROPERTIES LTD	ADRON HOMES PROPERTIES LTD	20,000,000.00
79 CWAY	CWAY	20,000,000.00
80 AHMADU MAHMOUD	AHMADU MAHMOUD	20,000,000.00
81 GROOMING PEOPLE FOR BETTER LIVELIHOOD	GROOMING PEOPLE FOR BETTER LIVELIHOOD	14,999,838.75
82 TETRA PAK (WEST AFRICA) LTD	TETRA PAK (WEST AFRICA) LTD	12,500,000.00
83 APAPA BULK TERMINAL	APAPA BULK TERMINAL	10,000,000.00
84 ECN TERMINAL	ECN TERMINAL	10,000,000.00
85 EKEOMA EME EKEOMA	EKEOMA EME EKEOMA	10,000,000.00
86 GREENWICH TRUST LIMITED	GREENWICH TRUST LIMITED	10,000,000.00
87 LADOL LOGISTICS LIMITED	LADOL LOGISTICS LIMITED	10,000,000.00
88 SIL CHEMICALS LIMITED	SIL CHEMICALS LIMITED	10,000,000.00
89 GREGORY PETER OBI	GREGORY PETER OBI	10,000,000.00
90 COWRY ASSET MANAGEMENT LIMITED	COWRY ASSET MANAGEMENT LIMITED	9,999,838.75
91 VISTA INTERNATIONAL	VISTA INTERNATIONAL	9,999,838.75
92 NIGERIAN TEXTILE MANUFACTURERS ASS.	NIGERIAN TEXTILE MANUFACTURERS ASS.	9,999,838.75
93 REMITA PLATFORM	REMITA PLATFORM	9,999,838.75
94 DRASA HEALTH TRUST	DRASA HEALTH TRUST	7,601,784.00
95 COMET SHIPPING AGENCIES NIGERIA	COMET SHIPPING AGENCIES NIGERIA	5,000,000.00
96 JENNIFER RAMATU ETUH FOUNDATION	HANDY CAPITAL LTD	5,000,000.00
97 MECURE INDUSTRIES	MECURE INDUSTRIES	5,000,000.00
98 DR AND MRS OKEY ORAMAH	DR AND MRS OKEY ORAMAH	5,000,000.00
99 ERISCO FOODS LIMITED	ERISCO FOODS LIMITED	5,000,000.00
100 DAMINA MAI ALBA	DAMINA MAI ALBA	4,700,000.00
101 JUBALI BROTHERS LIMITED	JUBALI BROTHERS LIMITED	4,000,000.00
102 NIGERIA BRITISH CHAMBER OF COMMERCE	NIGERIA BRITISH CHAMBER OF COMMERCE	4,000,000.00
103 DE ELITE IMPEX DISTRIBUTION CO LTD	DE ELITE IMPEX DISTRIBUTION CO LTD	3,000,000.00
104 ADG INTERNATIONAL RESOURCES LTD	ADG INTERNATIONAL RESOURCES LTD	2,500,000.00
105 NORRENBERGER INVESTMENT CAPITAL LTD	NORRENBERGER INVESTMENT CAPITAL LTD	2,000,000.00

106 OCEAN LORDS LIMITED	OCEAN LORDS LIMITED	2,000,000.00
107 AFRICAN NEWSPAPER OF NIGERIA PLC	AFRICAN NEWSPAPER OF NIGERIA PLC	1,666,250.00
108 OMNIK LIMITED	OMNIK LIMITED	1,199,516.25
109 FEW CHORE FINANCE COMPANY LTD	FEW CHORE FINANCE COMPANY LTD	1,000,000.00
110 PROSHARENA LIMITED	PROSHARENA LIMITED	1,000,000.00
111 M.E.C PROJECT LTD	M.E.C PROJECT LTD	1,000,000.00
112 RTGS INTERFACE	RTGS INTERFACE	1,000,000.00
113 EAC TRUSTEES	EAC TRUSTEES	1,000,000.00
114 INFINITY TRUST MORTGAGE BANK	INFINITY TRUST MORTGAGE BANK	1,000,000.00
115 WEST AFRICA SEASONING COMPANY LTD	WEST AFRICA SEASONING COMPANY LTD	1,000,000.00
116 REMITA PLATFORM	REMITA PLATFORM	999,838.75
117 DEEPLAST NIGERIA LIMITED	DEEPLAST NIGERIA LIMITED	999,838.75
118 CONTINENTAL BROADCASTING SERVICE	CONTINENTAL BROADCASTING SERVICE	911,364.58
119 TARABAROZ FISHERIES LIMITED	TARABAROZ FISHERIES LIMITED	501,451.25
120 ABAYOMI FOLORUNSHO	ABAYOMI FOLORUNSHO	500,000.00
121 CITITRUST GROUP	CITITRUST GROUP	500,000.00
122 ISI AVIATION LTD	ISI AVIATION LTD	499,838.75
123 REMITA PLATFORM	REMITA PLATFORM	499,838.75
124 REMITA PLATFORM	REMITA PLATFORM	399,838.75
125 REMITA PLATFORM	REMITA PLATFORM	299,838.75
126 SDMA NETWORK NIG. LTD	SDMA NETWORK NIG. LTD	201,236.25
127 KANAKALA LAKSHMIPATHI NAIDU	NAIDU KANAKALA	100,698.75
128 HIS MERCY SANCTUARY GLOBAL	HIS MERCY SANCTUARY GLOBAL	99,838.75
129 BABATUNDE KOLAWOLE	BABATUNDE KOLAWOLE	99,838.75
130 OLAGUNJU EBIERE	OLAGUNJU EBIERE	99,838.75
131 ANURAG DHIMAN	ANURAG DHIMAN	51,000.00
132 USMAN AHMED	USMAN AHMED	50,000.00
133 KAIROS HOF CONSULTANTS LTD	KAIROS HOF CONSULTANTS LTD	50,000.00
134 SATHYASUBHASH SUYAMBUDURAI	SATHYASUBHASH SUYAMBUDURAI	25,000.00
135 RAPHAEL OYOMITOLE NWANI	RAPHAEL OYOMITOLE NWANI	20,268.75
136 CHUKS EJECHI	CHUKS EJECHI	20,000.00
137 COGNITIVE SOLUTIONS PLC	COGNITIVE SOLUTIONS PLC	19,838.75
138 ANYAEHIE STANISLAUS IKECHUKWU	ANYAEHIE STANISLAUS IKECHUKWU	10,000.00
139 FREDERICK KIGHA	FREDERICK KIGHA	10,000.00
140 JAYAKUMAR SELVVAM	JAYAKUMAR SELVVAM	10,000.00

141 MANJI TABWAHAT LONGMUT	MANJI TABWAHAT LONGMUT	10,000.00
142 AYO OBE	AYO OBE	9,838.75
143 REMITA PLATFORM	REMITA PLATFORM	9,838.75
144 KES COMPUTER ENGINEERS LTD	KES COMPUTER ENGINEERS LTD	9,838.75
145 OLUKUNLE OLWASEGUN	OLUKUNLE OLWASEGUN	9,838.75
146 PAUL MADEBO	PAUL MADEBO	5,000.00
147 OLUMIDE SOYEMI	OLUMIDE SOYEMI	5,000.00
148 EJIMOFOR CHIJINDU	EJIMOFOR CHIJINDU	5,000.00
149 TOCHUKWU UMEH	TOCHUKWU UMEH	5,000.00
150 REMITA PLATFORM	REMITA PLATFORM	4,838.75
151 MR. & MRS. OJO EDWARD OLUWAJOBA	MR. & MRS. OJO EDWARD OLUWAJOBA	3,000.00
152 SUNDAY ISMAIL	SUNDAY ISMAIL	2,200.00
153 HAMZA MUHAMMAD KAMBA	HAMZA MUHAMMAD KAMBA	2,000.00
154 HELEN.O.GARBA	HELEN.O.GARBA	2,000.00
155 REMITA PLATFORM	REMITA PLATFORM	1,838.75
156 REMITA PLATFORM	REMITA PLATFORM	1,838.75
157 REMITA PLATFORM	REMITA PLATFORM	1,838.75
158 REMITA PLATFORM	REMITA PLATFORM	1,838.75
159 ADEWOLE ABIODUN	ADEWOLE ABIODUN	1,838.75
160 UGOCHUKWU MGBEAHURIKE	UGOCHUKWU MGBEAHURIKE	1,838.75
161 FATIMA ABDUL	FATIMA ABDUL	1,828.00
162 MICAH AYODEKE OLUWADUROTIMI	MICAH AYODEKE OLUWADUROTIMI	1,500.00
163 KAMALU AMINU	KAMALU AMINU	1,000.00
164 OLUWADAMILOLA	FAGBIYE	1,000.00
165 MOHAMMED GHALI MUHAMMED	MOHAMMED GHALI MUHAMMED	200.00
166 ADAMU YUSUF	ADAMU YUSUF	100.00
167 UCHENIDU COLLINS	UCHENIDU COLLINS	100.00
168 BASHIR AUWAL	BASHIR AUWAL	60.00
169 BILAL ABDULSALAM	BILAL ABDULSALAM	50.00
170 UMAR NAFIU USMAN	UMAR NAFIU USMAN	50.00
171 BASHIR SULAIMAN	BASHIR SULAIMAN	20.00
172 SANI ALTO ISAH	SANI ALTO ISAH	20.00
173 ABUBAKAR SHEHU	ABUBAKAR SHEHU	10.00
174 SANNI ZAKARIYYA	SANNI ZAKARIYYA	10.00
175 IBRAHUM MOHD BELLO	IBRAHUM MOHD BELLO	8.00

176	ALHAJI MALLAM MUSA	ALHAJI MALLAM MUSA			5.00
177	IDRIS MUHAMMAD	IDRIS MUHAMMAD			5.00
178	ABUBAKAR ISMAIL ABUBAKAR	ABUBAKAR ISMAIL ABUBAKAR			2.00
179	BELLO SHUAIBU	BELLO SHUAIBU			1.00
180	GH	GF			1.00
				Total -	**29,868,537,264.27-**
	**FX CONTRIBUTION TO CACOVID RELIEF FUND**		$		
1	LOHIA CHARITY FOUNDATION			1,000,000 -	380,000,000.00-
				**-**	**30,248,537,264.27-**
